# Benefits of a Primary Care Clinic Co-Located and Integrated in a Mental Health Setting for Veterans With Serious Mental Illness

**Published:** 2012-02-02

**Authors:** Paul A. Pirraglia, Emily Rowland, Wen-Chih Wu, Peter D. Friedmann, Thomas P. O'Toole, Lisa B. Cohen, Tracey H. Taveira

**Affiliations:** Systems Outcomes and Quality in Chronic Disease and Rehabilitation, Providence VA Medical Center; Systems Outcomes and Quality in Chronic Disease and Rehabilitation, Providence VA Medical Center, and the Alpert Medical School of Brown University, Providence, Rhode Island; Systems Outcomes and Quality in Chronic Disease and Rehabilitation, Providence VA Medical Center, and the Alpert Medical School of Brown University, Providence, Rhode Island; Systems Outcomes and Quality in Chronic Disease and Rehabilitation, Providence VA Medical Center, and the Alpert Medical School of Brown University, Providence, Rhode Island; Systems Outcomes and Quality in Chronic Disease and Rehabilitation, Providence VA Medical Center, and the Alpert Medical School of Brown University, Providence, Rhode Island; Systems Outcomes and Quality in Chronic Disease and Rehabilitation, Providence VA Medical Center, Providence, Rhode Island, and the University of Rhode Island, Kingston, Rhode Island; Systems Outcomes and Quality in Chronic Disease and Rehabilitation, Providence VA Medical Center, the Alpert Medical School of Brown University, Providence, Rhode Island, and the University of Rhode Island, Kingston, Rhode Island

## Abstract

**Introduction:**

Efficacy trials have shown that primary care co-located in the mental health setting improves the receipt of high-quality medical care among people with serious mental illness. We tested whether implementation of such a program affected health service use and cardiovascular risk factor control among veterans with serious mental illness who had previously demonstrated limited primary care engagement.

**Methods:**

We performed a cohort study of veterans enrolled in a co-located, integrated primary care clinic in the mental health outpatient unit through targeted chart review. Two successive 6-month periods in the year before and in the year following enrollment in the co-located primary care clinic were examined for primary care and emergency department use and for goal attainment of blood pressure, fasting blood lipids, body mass index (BMI), and, among patients with diabetes, hemoglobin A1c (HbA1c). We used repeated-measures logistic regression to analyze goal attainment and repeated measures Poisson regression to analyze service use.

**Results:**

Compared with the period before enrollment, the 97 veterans enrolled in the clinic had significantly more primary care visits during 6 months and significantly improved goal attainment for blood pressure, low-density lipoprotein cholesterol, triglycerides, and BMI. Changes with regard to goal attainment for high-density lipoprotein cholesterol and HbA1c were not significant.

**Conclusion:**

Enrollment in a co-located, integrated clinic was associated with increased primary care use and improved attainment of some cardiovascular risk goals among veterans with serious mental illness. Such a clinic can be implemented effectively in the mental health setting.

## Introduction

Cardiovascular disease (CVD) risk factors are common among patients with serious mental illnesses (SMI) such as schizophrenia, schizoaffective disorder, and bipolar disorder (1-7). The quality of care for CVD is poor in patients with SMI, and their CVD risk factors are commonly missed or ignored (8).

Veterans with SMI have fewer medical visits than do other US Veterans Administration (VA) patients (9). SMI patients primarily seek care for mental health conditions rather than for physical conditions (8), so the mental health setting may be a more effective "home" site for primary care services (10). Co-location and integration of primary care services in the mental health setting is an innovation that may reduce some of the barriers to delivery and receipt of high-quality medical care among patients with SMI (11,12). Co-location refers to the placement of primary care providers in the mental health setting, and integration is coordination of care with mental health providers (13). Previous studies of this care model have shown an increase in primary care visits, improved attainment of performance measures, and reduced emergency department use (11,14-17). However, these studies were limited in their ability to demonstrate that co-located care can be implemented in a clinical setting and to assess the effect of the clinic on CVD risk management. This is because these studies were done in an experimental setting, did not examine within-patient changes, or did not study the effect on cardiovascular measures.

We explored the effect of enrollment in a primary care clinic co-located and integrated in the outpatient mental health program on service use and control of CVD risk among veterans with SMI. We hypothesized that enrollment in this clinic would improve primary care access, reduce emergency department visits and hospitalizations, and improve control of CVD risk factors.

## Methods

### Serious Mental Illness Primary Care Clinic

The Serious Mental Illness Primary Care Clinic (SMIPCC) was implemented at the Providence VA Medical Center in March 2008. SMIPCC is a primary care clinic co-located and integrated in the mental health outpatient program. It is open 1 session per week and staffed by a single primary care provider and a patient care assistant. SMIPCC uses open-access scheduling. As much as possible, primary care visits coincide with scheduled mental health visits, although patients are sometimes asked to return at other times. Patients can also walk in for care. All patients seeking care are seen the same day.

To be enrolled in SMIPCC, a veteran must have a chronic and active mental health condition that leads to a high frequency of mental health service use. Veterans must have demonstrated poor access to primary care by having had at least 1 no-show or 2 "cancellations by patient" of a scheduled primary care visit in the prior 2 years; veterans not yet enrolled in primary care are also eligible. To support the care integration, the patient must have a mental health visit scheduled on the morning that SMIPCC is open or be enrolled in a mental health case management program that can assist with care coordination. The patient must have at least 1 concurrent medical diagnosis that is chronic and must agree to receive primary care through SMIPCC.

### Study population and design

We performed a longitudinal cohort study of all veterans enrolled in SMIPCC. The only inclusion criterion for the study was enrollment in SMIPCC for at least 1 year; there were no study exclusion criteria. Our study was approved by the Providence VA Medical Center institutional review board, and, because this was a chart review study with no direct patient contact, an informed consent waiver was granted.

### Data collection

We abstracted all data from electronic medical records. Demographic information at the time at which the patient initiated participation in SMIPCC included age, sex, race/ethnicity, marital status, comorbid medical and psychiatric conditions, and VA service connection. We treated age as a continuous variable. We categorized race/ethnicity as white non-Hispanic or not. Medical comorbidity was evaluated using the Charlson-Deyo comorbidity index (18). This score is calculated on the basis of the number of diseases (determined by using *International Classification of Diseases, 9th Revision* [ICD-9] codes), which are then weighted on the basis of 1-year risk of death; the sum of the weighted disease count is the score. Service connection is a rating the VA provides on the basis of degree of disability and association with military service that is used to determine the level of benefits for which a veteran is eligible from the VA. We classified VA service connection as not service connected, less than 50% service connected, or 50% to 100% service connected.

We used 4 observation windows: T1 and T2 were the 2 successive 6-month periods in the year before enrollment in SMIPCC, and T3 and T4 were the 2 successive 6-month periods of enrollment. T1 started exactly 1 year before enrollment in SMIPCC, T2 started 6 months before the date of enrollment, T3 started with the date of enrollment, and T4 started 6 months after the date of enrollment.

In each observation window, we collected data on blood pressure at scheduled outpatient visits (ie, not including emergency department or inpatient measurements), body mass index (BMI), low-density lipoprotein (LDL) cholesterol, high-density lipoprotein (HDL) cholesterol, triglycerides, and, among those with diabetes, hemoglobin A1c (HbA1c). For measures recorded more than once, we used the average value in the observation window.

Goal attainment outcomes were based on established performance measures (19). Goal blood pressure was systolic blood pressure less than 140 mm Hg and diastolic blood pressure less than 90 mm Hg; for patients with diabetes or coronary artery disease, goal blood pressure was less than 130 mm Hg systolic and 80 mm Hg diastolic. The goal for BMI, which was calculated by dividing the patient's weight by the square of the patient's height, was less than 30 kg/m^2^. Goal LDL cholesterol was less than 130 mg/dL, unless there was comorbid diabetes or coronary artery disease, in which case the goal was less than 100 mg/dL. Goal HDL cholesterol was more than 40 mg/dL for men and more than 50 mg/dL for women. Triglyceride goal was less than 150 mg/dL. Among patients with diabetes, goal HbA1c was less than 9%. For all measures, we considered missing data in an observation window to be not attained.

We examined health service use by using data from two 6-month observation windows, 1 for pre-enrollment (the 6-month period beginning exactly 1 calendar year before enrollment) and 1 for postenrollment (the 6-month period beginning 90 days after enrollment). We obtained the count of primary care visits to providers (physicians and nurse practitioners), emergency department visits (nonpsychiatric-related), and medical/surgical hospital admissions in these 6-month windows. We also recorded whether a primary care visit with a primary care provider was on the same day as a scheduled mental health visit of any type.

### Analyses

We used repeated measures logistic regression to examine the attainment of goal blood pressure, LDL and HDL cholesterol, triglycerides, BMI, and HbA1c, from pre-enrollment to postenrollment. To do this, the patient was considered to be the repeated effect, with time designated as a within-subjects factor. We specified a first-order autoregressive covariance structure based on the anticipated within-subject correlations with respect to time. We first examined the designation of pre-enrollment or postenrollment as the sole covariate in the fixed-effect portion of the model, then added age, sex, race/ethnicity, Charlson-Deyo comorbidity index score, and VA service connection to the fixed-effects models. We also examined actual measured values with a similar approach, using generalized linear models.

We performed several sensitivity analyses. We limited analysis to only windows T1 and T2 to test for temporal trends before enrollment. We examined our findings for attainment of goals, excluding patients with missing values (ie, treated as missing rather than not attaining goal). We repeated analyses using only patients who had transferred from usual primary care into SMIPCC (ie, excluded those who were new to VA primary care at the time of SMIPCC enrollment) to test whether transfer of primary care conferred a benefit. Finally, we focused only on patients with known coronary artery disease, diabetes, or both.

For service use data, we used repeated-measures Poisson regression. We first examined enrollment as a sole fixed effect, then added age, sex, race/ethnicity, Charlson-Deyo comorbidity index score, and VA service connection to the fixed-effects portion of the models. We used repeated measures logistic regression to examine the odds of a primary care visit concurrent with a mental health visit before and after enrollment. Few patients had a medical/surgical hospitalization in either observation window (1 in the 6 months before and 4 in the 6 months after enrollment), so we did not perform statistical tests on this measure. All analyses were performed using SAS version 9.1 (SAS Institute Inc, Cary, North Carolina), and significance was set at *P* < .05.

## Results

Most veterans in our study (N = 97) were male and non-Hispanic white, and mean age was 55.3 years (range, 28-86 y) (Table 1). The median Charlson-Deyo comorbidity index was 1, with an interquartile range (IQR) of 2 (0-2); 10% of the population had a score of 3 or more.

### Goal attainment

In the repeated-measures logistic regression models, enrollment in SMIPCC was associated with higher goal attainment for blood pressure (adjusted odds ratio [AOR] = 2.16; 95% confidence interval [CI], 1.47-3.18), LDL cholesterol (AOR = 1.60; 95% CI, 1.10-2.34), triglyceride (AOR = 1.64; 95% CI, 1.06-2.51), and BMI (AOR = 1.81; 95% CI,1.29-2.54) (Figure). No significant difference was found for goal HDL cholesterol or HbA1c. There were no differences between measured values across observation windows (Table 2), but the number of patients for whom the measure was obtained was lower in windows T1 and T2 (ie, before enrollment).

**Figure.**
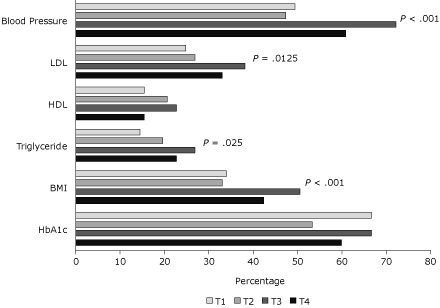
Percentage of patients attaining cardiovascular disease risk goals at selected observation windows of the Serious Mental Illness Primary Care Clinic (SMIPCC). Windows T1 and T2 were the 2 successive 6-month periods in the year before enrollment in SMIPCC, and T3 and T4 were the 2 successive 6-month periods of enrollment. *P* values represent significant differences between pre-enrollment and postenrollment in fully adjusted repeated measures logistic regression analyses. See Methods for descriptions of laboratory values that consitute goal attainment. Abbreviations: LDL, low-density lipoprotein; HDL, high-density lipoprotein; BMI, body mass index; HbA1c, hemoglobin A1c.

**Table:** 

**Observation Window**	**Blood Pressure**	**LDL Cholesterol**	**HDL Cholesterol**	**Triglyceride**	**BMI**	**HbA1c**

	**%**					
T1	49.6	24.7	15.5	14.4	34.0	66.7
T2	47.4	26.8	20.6	19.6	33.0	53.3
T3	72.2	38.1	22.7	26.8	50.5	66.7
T4	60.8	33.0	15.5	22.7	42.3	60.0

Results of goal attainment analyses limited to T1 and T2 to test for temporal change in the period before enrollment were not significant, nor were findings for attainment of goals excluding subjects with missing values. Repeated-measures logistic regression analyses of only patients who had transferred usual primary care to SMIPCC showed that the adjusted models still had significant findings for blood pressure (AOR = 1.22; 95% CI, 1.15-2.50; *P* = .01), LDL cholesterol (AOR = 1.22; 95% CI, 1.01-2.18; *P* = .04), and BMI (AOR = 1.19; 95% CI, 1.26-2.51; *P* = .01). Among the 28 veterans with coronary artery disease, diabetes, or both, repeated measures logistic regression models showed enrollment was associated with a significant improvement in blood pressure goal attainment (AOR = 1.32; 95% CI, 1.22-3.60; *P* = .01) but not with the other measures.

### Service use

Median number of primary care visits in the 6 months of observation before enrollment was 0, with an IQR of 1 (0-1) and overall range of 0 to 6. Median number of primary care visits in the 6-month observation window after enrollment was 2, with an IQR of 2 (1-3) and overall range of 0 to 12. Before enrollment, the median 6-month number of emergency department visits was 0, with an IQR of 1 (0-1) and overall range of 0 to 14; after enrollment the median 6-month number of emergency department visits was 0, with an IQR of 1 (0-1) and overall range of 0 to 6. Repeated-measures Poisson regression models showed that the number of primary care visits increased significantly after enrollment in SMIPCC (adjusted count = 3.4; 95% CI, 2.5-4.8; *P* < .001) compared with the number before enrollment, but the change in the number of emergency department visits was not significant.

In the observation window before enrollment, 49% of primary care visits with a provider were on the same day as any scheduled mental health visit, and this increased to 86% in the postenrollment observation window. Compared with the pre-enrollment period, repeated measures logistic regression analysis showed the odds of a primary care visit concurrent with a mental health visit was 7.13 (95% CI, 3.26-15.6; *P* < .001).

## Discussion

Veterans with SMI had improved attainment of CVD risk factor goals after being enrolled in a primary care clinic co-located and integrated into the outpatient mental health clinic. Our findings are consistent with those of other reports in the literature regarding the benefits of co-location on quality of care and access (12). In the VA, the efficacy of a co-located, integrated primary care program in the mental health setting was demonstrated in a randomized controlled trial (11). Another study reported higher attainment of blood pressure and LDL cholesterol goals, but it compared the SMI population to the general population rather than examining change in these measures in the SMI population (14). Outside the VA, medical care management for patients with SMI in community mental health centers has been effective (17), and researchers of this study observed a decrease in Framingham Risk Score among participants with laboratory values. However, Framingham Risk Score has been reported to be less reliable among patients with lower socioeconomic status (20), which may comprise a large portion of patients in community mental health centers. Our findings add to this body of work because, in data from a nonexperimental population examined before and after enrollment in the co-located clinic, we examined measurements of individual risk factors (blood pressure and fasting lipid panel in particular) rather than a composite score and demonstrated greater attainment of goals for these risk factors.

We observed a higher rate of primary care use, consistent with previous studies of this model of care. The rate of emergency department visits was not significant, but this finding may have been due to lack of power (the lower limit of the 95% CI was 0.91). Our findings regarding service use demonstrate the responsiveness of this model of care to patient need, particularly as the clinic is open access. Recent work has suggested that co-location of general medical services in the mental health setting reduces ambulatory care sensitive hospital admissions (21), which are potentially preventable with quality primary care delivery (22). The rate of hospitalizations was too low to examine in this study.

We note that the measured values did not change across the observation windows, and the measured values were generally good. This finding implies that the primary benefit from enrollment was in obtaining measurements in patients without prior measurements. Of note, 13% of the 97 patients enrolled in SMIPCC were new to primary care. Most patients had been enrolled in usual primary care previously, suggesting that the clinic effectively addressed low engagement in primary care, as intended. The VA considers missing performance measures as not at goal, so the finding of improvements in goal attainment is relevant from this perspective.

Patients with SMI may not receive optimal care because of organizational barriers and limited communication between their primary care and mental health providers (23). Drapalski et al found that 60% of veterans with SMI perceived barriers to access to medical care, and among the barriers, personal factors were the most common (24). We speculate that our clinic had a positive effect on control of CVD risk factors and service use because it addressed organizational and personal barriers to care by being convenient, patient-centered with open-access appointments, and linked to mental health service delivery. The co-location and linkage to mental health service delivery were key aspects to promoting integration, as was the proximity of primary care to mental health providers. Furthermore, the tandem nature of primary care and mental health visits promoted communication between primary care and mental health providers as well as between these providers and patients, which was evident in the concurrence between primary care and mental health visits in the postenrollment period.

Limitations of this study include constraints on the ability to generalize outside the VA and lack of an economic analysis. Our study was conducted at 1 site, so we cannot generalize beyond it. The VA health care system itself, as well as the population it serves, may be unique. We used a pre/post design, and therefore lack concurrent controls. However, examination of the 2 periods before enrollment showed no change at all, suggesting the effect we saw from pre- to postenrollment was attributable to the clinic rather than temporal trends. The cost-benefit of such a clinic would be valuable information, because a high-cost intervention would not be appealing, even if it were effective; such analysis is beyond the scope of the work we present here. However, previous work has reported that co-located, integrated programs are cost-neutral (11,12).

In summary, our primary care clinic for veterans with SMI that was co-located and integrated in the mental health setting improved attainment of CVD risk factor goals and increased primary care use. Our study demonstrated that the effects observed in efficacy studies of this model of care hold in a real-world clinic, supporting the concept that co-located, integrated primary care clinics can be implemented successfully in the mental health setting. Future studies of primary care for patients with SMI integrated into the mental health setting should determine best practices (ie*,* clinic structure, staffing, practices, and population management) and costs to better understand the facilitators and barriers to successful implementation.

## Figures and Tables

**Table 1.** Patient (N = 97) Demographic and Clinical Characteristics, Serious Mental Illness Primary Care Clinic, Providence, Rhode Island, 2008

**Characteristic^a^**	n (%)^b^
Male sex	92 (95)
Non-Hispanic white race/ethnicity	83 (86)
VA service–connected disability >50%	40 (41)
Diabetes^c^	15 (15)
Dementia^c^	3 (3)
Liver disease, mild to moderate^c^	19 (20)
Liver disease, severe^c^	1 (1)
Renal disease^c^	6 (6)
Congestive heart failure^c^	7 (7)
Myocardial infarction^c^	4 (4)
Peripheral artery disease^c^	2 (2)
Stroke^c^	2 (2)
Chronic obstructive pulmonary disease^c^	12 (12)
Peptic ulcer disease^c^	7 (7)
Autoimmune connective tissue disease^c^	1 (1)
Cancer without metastasis^c^	14 (14)
Cancer with metastasis^c^	1 (1)
Hyperlipidemia	60 (62)
Hypertension	45 (46)
Coronary artery disease	15 (15)
Schizophrenia	23 (24)
Schizoaffective disorder	24 (25)
Psychosis, not otherwise specified	4 (4)
Bipolar disorder	14 (14)
Major depressive disorder	36 (37)
Alcohol abuse/dependence^d^	41 (42)
Substance abuse/dependence^d^	28 (29)

a Medical and psychiatric conditions are not mutually exclusive. VA service-connected disability refers to the VA's rating of degree of disability related to military service.

b Mean age was 55.3 y (standard deviation, 10.0 y).

c Denotes conditions used in the determination of the Charlson-Deyo Comorbidity Index.

d Alcohol and substance abuse/dependence includes both past and current.

**Table 2.** Cardiovascular Disease Risk Measurement Values in Each Observation Window,^a^ Serious Mental Illness Primary Care Clinic, Providence, Rhode Island, 2008^b^

Measure	T1		T2		T3		T4	

	n^c^	Mean (SD)	n^c^	Mean (SD)	n^c^	Mean (SD)	n^c^	Mean (SD)
SBP, mm Hg	61	125.2 (12.9)	59	122.3 (14.6)	95	125.4 (15.3)	71	125.2 (16.0)
DBP, mm Hg	61	75.5 (10.2)	59	73.6 (9.7)	95	76.4 (9.6)	71	74.9 (8.6)
LDLC, mg/dL	42	114.7 (34.8)	39	105.2 (36.1)	56	114.0 (31.5)	42	99.2 (33.9)
HDLC, mg/dL	42	40.8 (12.3)	38	41.9 (17.1)	56	41.9 (14.9)	42	40.9 (5.3)
TG, mg/dL	41	236.1 (187.6)	38	191.0 (200.7)	57	206.4 (165.8)	42	190.2 (134.6)
BMI, kg/m^2^	69	30.8 (6.7)	65	30.3 (6.4)	96	30.2 (6.3)	77	29.6 (5.8)
HbA1c, %	11	7.1 (1.4)	9	7.2 (2.0)	10	6.7 (0.7)	10	6.8 (1.2)

Abbreviations: SD, standard deviation; SBP, systolic blood pressure; DBP, diastolic blood pressure; LDLC, low-density lipoprotein cholesterol; HDLC, high-density lipoprotein cholesterol; TG, triglycerides; BMI, body mass index; HbA1c, hemoglobin A1c.

a The T1 window was 12 to 6 months before enrollment; T2 was 6 months to enrollment; T3 was enrollment date to 6 months postenrollment; and T4 was 6 to 12 months postenrollment.

b No significant differences were found between observation windows.

c n = number of patients with observations.
